# Supplementary material for: Association between oral microbiome and seven types of cancers in East Asian population: a two-sample Mendelian randomization analysis
Source: Front Mol Biosci. 2023 Nov 21;10:1327893. doi: 10.3389/fmolb.2023.1327893 (PMC10702768; doi:10.3389/fmolb.2023.1327893)
Supplement: Supplementary file 1 [file DataSheet1.ZIP › Supplementary Materials/MR plots for tongue/tongue═╝/Ovarian cancer/pheno.1948_to_ovarian cancer_forest.pdf]

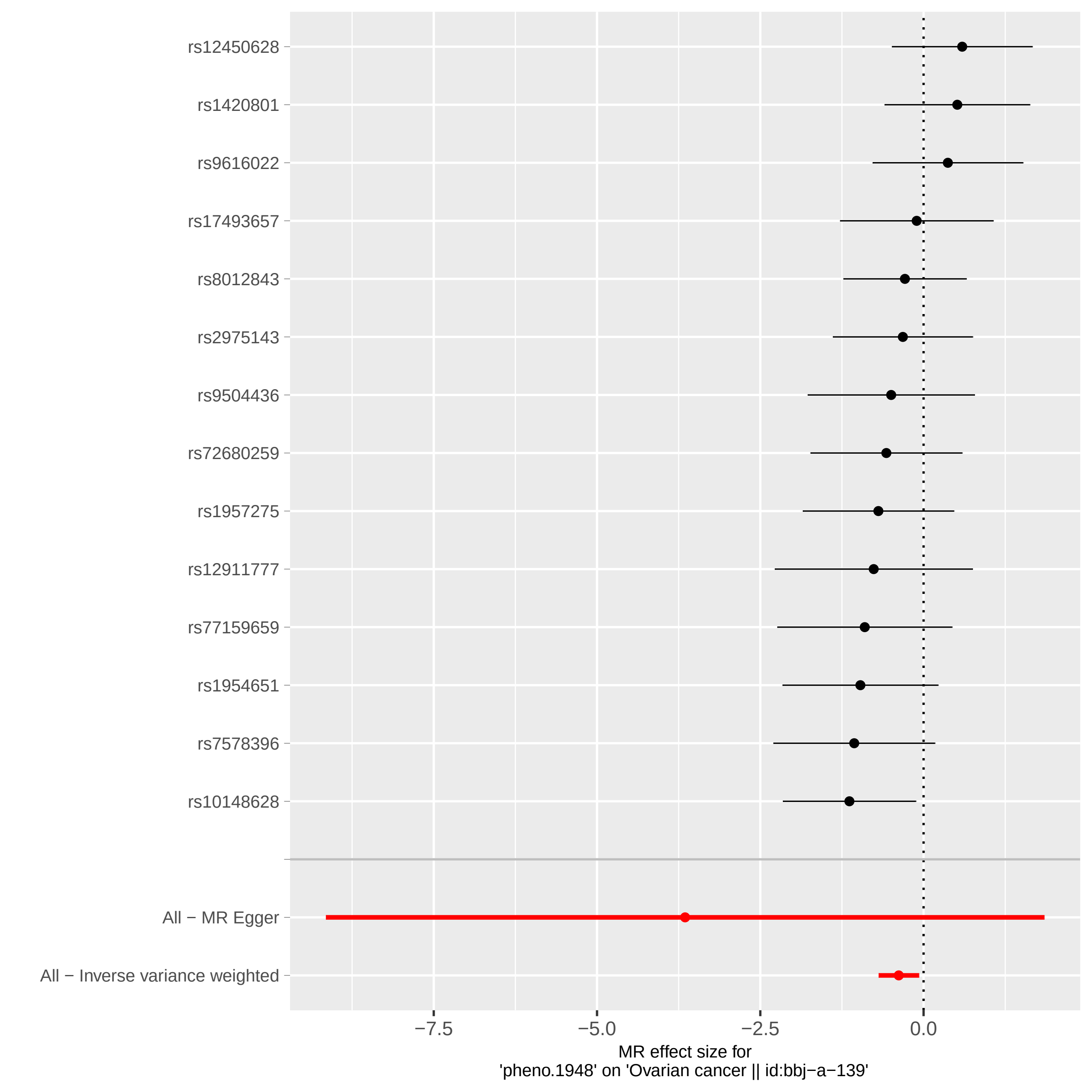

rs12450628
rs1420801
rs9616022
rs17493657
rs8012843
rs2975143
rs9504436
rs72680259
rs1957275
rs12911777
rs77159659
rs1954651
rs7578396
rs10148628
All − MR Egger
All − Inverse variance weighted
−7.5
−5.0
−2.5
0.0
MR effect size for
'pheno.1948' on 'Ovarian cancer || id:bbj−a−139'
